# Proximate composition and fatty acid analysis of *Lablab purpureus* (L.) legume seed: implicates to both protein and essential fatty acid supplementation

**DOI:** 10.1186/s40064-016-3587-1

**Published:** 2016-10-28

**Authors:** Shahdat Hossain, Rashed Ahmed, Sujan Bhowmick, Abdullah Al Mamun, Michio Hashimoto

**Affiliations:** 1 Department of Biochemistry and Molecular Biology, Laboratory of Alternative Medicine and Behavioral Neurosciences, Jahangirnagar University, Savar, Dhaka, 1342 Bangladesh; 2 Department of Environmental Physiology, Faculty of Medicine, Shimane University, Izumo, Shimane 693-8501 Japan

**Keywords:** *L. purpureus*, Proximate composition, Omega-3 and omega-6 polyunsaturated fatty acid

## Abstract

The high mortality rate in Bangladesh is related to poverty, which results in protein malnutrition, essential fatty acid deficiency and lacks in adequate vitamins, minerals and calorie. Exploring new food items with improved dietary nutrition factors may, therefore, help to decrease the mortality rate in the poor countries like Bangladesh. Accordingly, the present study was a proximate composition and fatty acid analysis of *L. purpureus* seed—a legume seed which is given no careful attention locally, though it might be a good source of valuable nutrition factors for both animals and humans. The purpose of the study was, therefore, to generate awareness that *L. purpureus* could also act as a good source of food components essential for good health. Proximate analysis revealed that the seed powder contained 8.47 ± 0.52% moisture; 3.50 ± 0.0.07% ash; 1.02 ± 0.06% total fat; 23.95 ± 0.15% total protein; 1.21 ± 0.16% total dietary fiber; 61.86 ± 0.70% total carbohydrate and 352.4 ± 2.66 kcal/100 g energy. Phytic acid content (%) was 1.014 ± 0.048. Major fatty acid composition (%): the essential fatty acid linoleic acid (C18:2, ω-6) was 9.50 ± 0.68, while the linolenic acid (C18:3, ω-3) was 1.95 ± 0.18. Palmitic acid (C16:0), stearic acid (C18:0) and oleic acid (C18:1) were, respectively, 2.96 ± 0.19, 0.77 ± 0.04 and 1.10 ± 0.06. Lignoceric acid (C24:0) was 0.11 ± 0.007%. Monounsaturated palmitoleic acid (0.006 ± 0.0), docosapentaenoic acid (DPA, C22:5, ω-3) and nervonic acid (0.002 ± 0.0) were present in trace amounts. Arachidonic acid (AA, C20:4, ω-6), eicosapentaenoic acid (C20:5, ω-3), and docosahexaenoic acid (C22:6, ω-3) were not detected. The fatty acid profile, thus, suggests that essential omega-6 fatty acid linoleic acid (C18:3, ω-6) and omega-3 linolenic acid (C18:3, ω-3) were the major polyunsaturated fatty acids (PUFA) present in *L. purpureus* seed. In addition, the seed contained high amount of proteins. Finally, these results suggest that *L. purpureus* seed could be used as a good source of quality food components, including protein and essential fatty acids.

## Background

There is a pressing need in developing/poor countries like Bangladesh for alternative food sources that would be readily available, affordable and, at the same time, rich in energy and essential nutrients. Attention, in this regard, is now being paid on neglected and underutilized crops, commonly described as orphan crops. Our present study was, accordingly, concerned with such a neglected crop (*L. purpureus*) in Bangladesh, which has the full potential to be used as a good source of essential food components. *L. purpureus* (L.), also known as lablab bean or hyacinth bean, is a common bean belonging to the Leguminosae (Fabaceae) family; it originated in Africa and now is cultivated throughout the tropics including Bangladesh for its edible beans. In Bangladesh, various types of hyacinth bean are grown in different parts of the country with various popular local names (Islam et al. 2010). There are many genotype varieties with different morphotypes of *L. purpureus* in Bangladesh, with IPSA1, BARI Seam1 and Kartica varieties being the most abundant. They are commonly known as deshi seam. Lablab beans have both nutraceutical and pharmaceutical traits for use as a medicinal food (Morris 2009). Studies on nutrient composition showed that the bean is a good source of protein, carbohydrate and energy. The levels of protein in lablab beans are 20–25% (Akpapunam 1996; Karachi 1997; Elamin et al. 2013; Shaahu et al. 2015). While their lipid content is only 1.2% (El Hardallo et al. 1980), the total carbohydrates in the seeds, however, varied from 54 to 63% (Deaka and Sarkar 1990). Furthermore, the lablab seeds are also rich in the essential amino acids, such as lysine (Deaka and Sarkar 1990) and leucine (Kala et al. 2010). The lablab lipid also contains essential fatty acids such as linoleic acid (C18:2, ω-6) and alpha-linolenic acid (C18:3, ω-3), as reported by Kala et al. (2010). They contain 4.4–9.6% crude fibers (Dougall and Bogdan 1966; Kuo 1967; El Hardallo and El Tiny 1985). Micronutrients and minerals are present in the lablab seed (Kala et al. 2010; Shaahu et al. 2015). These reports obviously acknowledge that lablab bean is not only a rich source of proteins but also of other nutritive factors including essential fatty acids, starch, minerals and vitamins. They also contain important health protective compounds, such as phenolics, inositol phosphates and oligosaccharides (Ramadoss and Shunmugam 2014). The bean is, however, paid no careful attention and is mostly neglected in Bangladesh, excepting some street vendors who sell the fried seeds as a traditional afternoon snack. Dried seeds are also cooked or processed into cakes and eaten in some poor parts of Bangladesh as an alternative food item without knowing anything else about its nutrition quality. In spite of the nutritional supports they offer, beans, in general, have a very poor image in Bangladesh as a food source. They are called the “poor man’s meat,” which is consistent with the inverse relation between bean intake and income. The lablab seed is less known in the context of the fact that though it can provide many nutritional supports and thus could be beneficial, however, it is not being used to its full potential in many poor areas of the world including Bangladesh. Therefore effort must be devoted to conducting more research to extend both technical and practical knowledge about lablab so that its full potential may be achieved. For instance, besides its high protein content, other nutrients such as essential fatty acids of *L. purpureus* seed could also be considered of significance for the poor people of Bangladesh, where protein malnutrition and essential fatty acid deficiency is an existing reality. Polyunsaturated fatty acids (PUFAs) such as omega-6 linoleic acid (C18:2, ω-6) and omega-3 alpha-linolenic acid (C18:3, ω-3) are essential for humans, since they cannot be synthesized in the body and must, therefore, be ingested through foods. The highly unsaturated ω 3-polyunsaturated fatty acid (PUFA) docosahexaenoic acid (DHA, C22:6, ω-3), which is directly obtained from fish and other sea-based animals and/or from precursor omega-3 α-linolenic acid of plant origin, is essential for normal growth and development and may play an important role in the prevention and treatment of coronary artery disease, hypertension, diabetics, arthritis, other inflammatory and autoimmune disorders and cancer (Wang and Jones 2004). We, thus, consider that *L. purpureus* seed, as a legume, may also represent, at least partially, a source of ω-3 and/or ω-6 essential fatty acids important for human nutrition, in particular for the vegetarians, and other mammals, where the consumptions of PUFAs such as docosahexaenoic acid (DHA, C22:6, ω-3) is extremely limited. Besides, *L. purpureus* is among the vegetables in which the proximate and fatty acid analyses have not been extensively studied in Bangladesh. Therefore, the present investigation was carried out with an attempt to clarify the proximate chemical composition as well as fatty acid profile of *L. purpureus* seeds.

## Methods

### Chemicals

DHA-95E, an ethyl all *cis*-4,7,10,13,16,19-docosahexaenoic acid, was obtained from Harima Chemicals (Tokyo, Japan). Tricosanoic acid, linoleic acid, α-linolenic acid, arachidonic acid, eicosapentaenoic acid and other fatty acids, phytic acid, bipyridine were purchased from Sigma Chemical Co. (St Louis, MO, USA). All other materials of the highest grade were obtained commercially.

### Collection of *L*. *purpureus* seeds

**Fig. 1:**
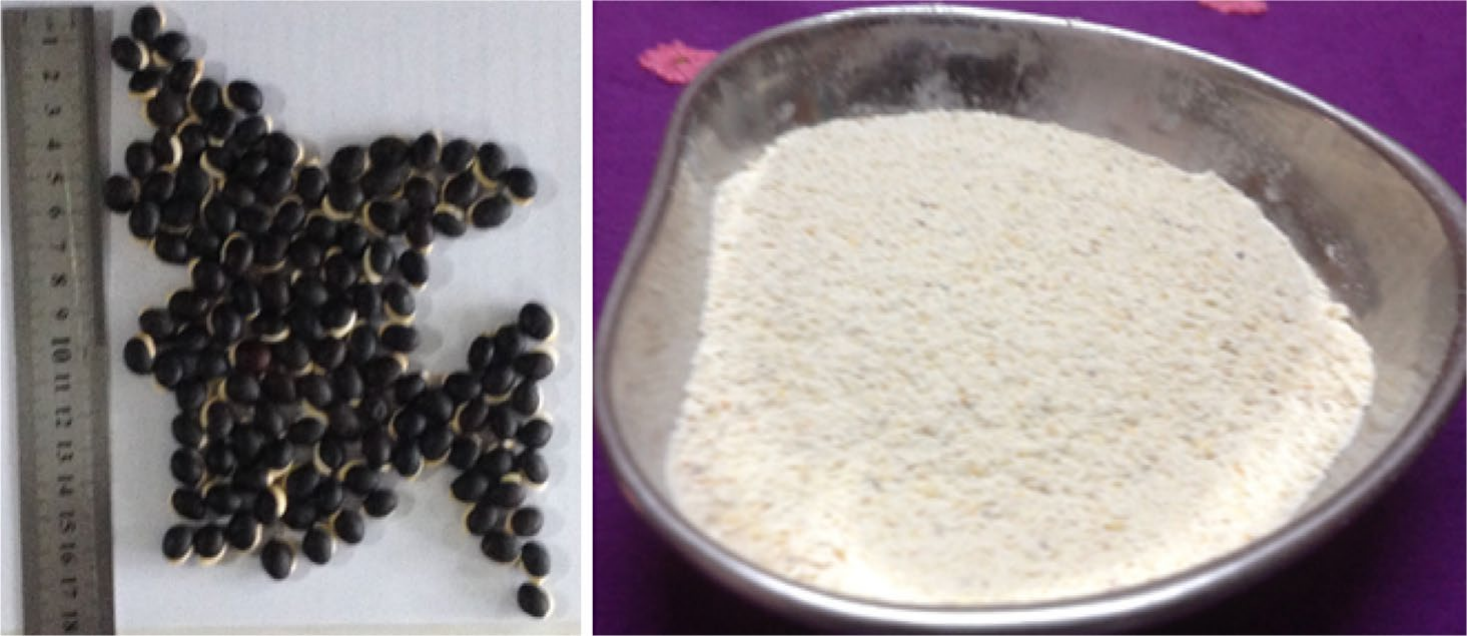
*L. purpureus* seed and seed powder used in the investigation

The country bean variety (*Lablab purpureus* L.), namely BARI Seam 1 seeds were collected from local market and authenticated by a Botanist of the Department of Botany, Jahangirnagar University, Savar, Dhaka. The length of the dry seeds was 11 ± 0.90 mm and the width was 8.02 ± 0.78 mm, while 1 kg of *L. purpureus* seed comprised ~1693 seeds. The sun dried seeds were powdered using mechanical grinder. The ground seed powder was then utilized for proximate composition, fatty acid profile and phytic acid analysis (Fig. 1).

### Proximate composition

The moisture content was determined by drying 20 g of *L. purpureus* seed in an oven at 105 °C for 24 h, according to the standard procedures detailed by AOAC (Association of Official Analytical Chemists 2005; Method # 925.09), and the result is expressed on a percentage basis. The air dried samples were powdered in a mill to 40-mesh size and stored in screw-capped bottles at room temperature for further analyses. The nitrogen content (N) was determined by Kjeldahl method, as described by AOAC (Association of Official Analytical Chemists 2005; Method # 920.152) and the crude protein content was calculated by multiplying the N with 6.25 (N × 6.25). Crude lipid content was determined using Soxhlet apparatus according to the method described by AOAC (Association of Official Analytical Chemists 2005; Method # 920.85). Available carbohydrate was estimated indirectly by difference (Food and Agriculture Organization, Food and Nutrition paper, 77, 2003). The ash content was determined by heating 2 g of the dried sample in a silica dish at 600 °C for 6 h, using the method described by AOAC (Association of Official Analytical Chemists 2005; 923.03). Total energy was also determined by the calculation of energy values of carbohydrate, fat, protein and crude fiber.

Total dietary fiber (TDF) was determined by non-enzymatic gravimetric method (Li and Cardozo 1994). To determine total TDF, duplicate 500 mg powder samples were taken in separate 250 ml beakers. To each beaker 25 ml water was added and gently stirred until the samples were thoroughly wetted, (i.e. no clumps were noticed). The beakers were covered with aluminum foil and allowed to stand 90 min without stirring in an incubator maintained at 37 °C. Afterwards, 100 ml 96% ethanol was added to each beaker and allowed to stand for 1 h at room temperature. The residue was collected under vacuum in a pre-weighed crucible containing filter aid. The residue was washed successively with 20 ml of 78% ethanol, 10 ml of 95% ethanol and 10 ml acetone. One crucible containing the residue was dried ≥2 h at 105 °C, and then cooled >2 h in a desiccator and weighed. One crucible containing the residue was used for ash determination at 525 °C for 5 h. The ash-containing crucible was cooled for >2 h in a desiccator and weighed. The residue from the remaining duplicate crucible was used for the protein determination by the Kjaldhal method as already mentioned. The TDF was calculated as follows:

$$ {\text{TDF\% }} = 100 \times \frac{{{\text{Wr}} - [({\text{P}} + {\text{A)/100}}]{\text{Wr}}}}{\text{Ws}} $$

where, Wr is the mg residue, P is the % of protein in the residue; A is the ash in the residue, and Ws is the mg sample. The nitrogen free extract was obtained by difference.

### Fatty acid profile

Fatty acid composition of the *L. purpureus* (L) legume seed powder was determined using a modification of the one-step reaction of Lepage and Roy (1986), as previously described (Hossain et al. 1998). To 10 mg of *L. purpureus* seed powder, 2.0 ml methanol-n-octane (4:1, v/v) containing 10 μg tricosanoic acid as internal standard and 200 μl acetyl chloride were added. The mixture was incubated at 100 °C for 60 min and cooled, then neutralized with 0.5 N aqueous NaOH containing 10% sodium chloride. The neutralized mixture was shaken for 10 min at room temperature and centrifuged at 180 °C for 5 min. The octane phase with the fatty acid methyl esters was directly subjected to gas chromatography. The gas chromatography separation was done on a Model 5890II (Hewlett–Packard, Avondale, PA, USA) equipped with a flame ionization detector and an automatic sampler Model 7673. A 30 m × 0.25 mm capillary column (DB-WAX P/N 122-7032, J & W Scientific, CA, USA) was initially maintained at 100 °C for 1 min, raised to 180 °C at 20 °C/min, then raised to 240 °C at 28 °C/min, further raised to 260 °C at 4 °C/min and maintained for 5 min. Peaks were identified by comparison with authenticated standards, quantified by peak area integration and expressed as weight percentage of total methyl esters, the relative weight percentage of each fatty acid was determined from integrated peak areas.

### Determination of phytic acid

The phytic acid was analyzed in the protocol as described by Haug and Lantzsch (1983). The seed samples were grounded and incubated at 70 °C for 3 days. The phytic acid extraction was carried out by adding 10 ml 0.2 N HCl to 0.5 g seed powder and placed on the horizontal shaker at room temperature for 24 h. Two ml of ferric ammonium sulfate solution was added to the 1 ml of an aliquot of the extract. The mixture of this solution was boiled at 100 °C for 30 min and left it at room temperature. This solution was then centrifuged at 5000 rpm at 4 °C for 15 min. Two ml of this supernatant was mixed with 3 ml of 2,2′-bipyridine solution with a vortex. After the mixing, the mixture solution was incubated for 30 min and the light absorbance was measured with a spectrophotometer at 530 nm.

### Statistical analysis

Mean ± SEM (Standard error of mean) were determined for all nutrients under present study. In addition, the coefficient of variation [CV % = (Standard deviation/Mean) × 100] is also shown in the Tables. Statistical analysis was accomplished by using Statistical Package for Social Sciences (SPSS/PC; Version12.0; SPSS Inc., Chicago).

## Results and discussion

Tables 1 and 2 represent data on proximate composition and fatty acid profile of *L. purpureus* seed powder respectively. *L. purpureus* seed powder contains noticeably higher values of total protein, total fat, total dietary fiber, ash content and gross energy and low in moisture content. The seed powder contained 8.47 ± 0.52% moisture, which is somehow intermediate when compared with the moisture values of other legumes ranging between 5.0 and 11% as reported in the previous studies (Lge et al. 1984; Aremu et al. 2006). The observed moisture content in the seeds is an indication that the activity of the microorganisms would be reduced and thereby might increase the shelf-life of the *Lablab* beans. This observation is also in agreement with the reports of Adeyeye and Ayejuyo (1994) and Olitino et al. (2007). The total ash content in *L. Purpureus* is 3.50 ± 0.07%. High total ash content signifies the presence of high levels of macro- and micro-minerals. The total protein content of *L. purpureus* was 23.95 ± 0.16%. The protein content of *L. purperus* is higher than the commonly consumed legume *Cicer arietinum* (Srivastava and Ali 2004; Khatoon and Prakash 2006). The remarkably high level of protein in the bean under study underscores its importance as a source of this vital nutrient to overcome the protein deficiency among the malnourished people of Bangladesh. Total fiber content in the seed was 1.21 ± 0.16%. The fiber content may improve bowel function and provide fecal bulk. The caloric value of *L. purpureus* seed powder was 352.4 K cal/100 g, indicating that it may be a great source for daily energy requirements. The phytic acid content in the seed powder 1.014 ± 0.048 (CV% 11.83). The phytic acid content in this study was comparable to those of the studies conducted by others, such as Shaahu et al. (2015), which indicated that the phytic acid contents in different cultivars of *L. purpureus* were in the range of 400–1200 mg per 100 gm dry seed powder. The total fat content of *L. purpureus* seed was found to be 1.02 ± 0.06%, suggesting that the seed can act, at least partially, as a source of quality vegetable oil (in terms of essential fatty acids) at least for the poor people who cannot include edible oil in their daily cooking, and also for cattle and other domestic animals.

**Table 1:** Proximate chemical analysis of *L. purpureus* L. seed powder (g/100 g)

Number of samples	Percentage (%)						Energy (Kcal)
6	Moisture	Ash	Fat	Protein	TDF	~CHO	352.42 ± 2.66
	8.47 ± 0.52	3.50 ± 0.07	1.02 ± 0.06	23.95 ± 0.16	1.21 ± 0.06	61.86 ± 0.79	
CV (%)	15.04	5.09	14.06	1.59	12.68	3.01	2.40

All values are expressed as mean ± SEM (standard error of mean). *TDF* total dietary fiber. ~CHO: Carbohydrate; CV (%) = (Standard deviation/mean) × 100

**Table 2:** Fatty acid profile (as % of total share) of the *L. purpureus* seed powder (mg/g dry powder)

	Mean ± SEM	CV (%)
PLA	2.96 ± 0.3428	11.58
POA	0.006 ± 0.0004	6.75
STA	0.76 ± 0.0659	8.6
OLA	1.1 ± 0.099	8.94
LLA	9.5 ± 1.1865	12.49
LnA	1.95 ± 0.3132	16.06
AA	ND	0
EPA	ND	0
DPA	0.003 ± 0.0003	11.37
LGN	±0.013	11.67
DHA	ND	0
NVA	0.002 ± 0.0002	10.74
USI	1.58 ± 0.0146	0.93

Results are mean ± SEM, for triplicate determinations. *PLA* palmitic acid (C16:0), *POA* palmitoleic acid (C16:1, ω-9); *STA* stearic acid (C18:0); *OLA* oleic acid (C18:1, ω-9); *LLA* linoleic acid (C18:2, ω-6), *LnA* linolenic acid (C18:3, ω-3); *AA* arachidonic acid (C20:4, ω-6), *EPA* eicosapentaenoic acid (C20:5, ω-3); *DPA* docosapentaenoic acid (C22:5, ω-3); *LGN* lignoceric acid (C24:0); *DHA* docosahexaenoic acid (C22:6, ω-3) and *NVA* nervonic acid (C24:1, ω-9). USI (Unsaturation Index) = ∑(mol% of each (poly)unsaturated fatty acid × number of double bond(s) per (poly)unsaturated fatty acid)/100. *ND* not detected. CV(%) = (Standard deviation/mean) × 100

The fatty acid composition of *L. purpureus* seed powder was determined by gas chromatography.

The *L. purpureus* bean powder was very low in total fat (1.10%) and its contribution to the total calorie was only ~2.8%. The predominant polyunsaturated fatty acids (PUFAs) in the *L. purpureus* bean was ω-6 linoleic acid (~58%), although the bean also contained ~12.0% ω-3 PUFA α-linolenic acid. Other major fatty acids accounted for: 18% palmitic acid; 4.67% stearic acid, 6.7% oleic acid. Palmitoleic acid, arachidonic acid, lignoceric acid and nervonic acid were present in trace amounts. Though the overall fat content of *L. purpureus* bean was low, the consumption of *L. purpureus* bean however can contribute significantly to α-linolenic acid intake. The dietary α-linolenic acid (LnA, C18:3, ω-3) might act as the precursor for highly polyunsaturated ω-3 fatty acids, including eicosapentaenoic (EPA, C20:5, ω-3) and docosahexaenoic acid (DHA, C22:6, ω-3). The dietary linoleic acid (LLA, C18:2, ω-6), on the other hand, might act as the precursor of arachidonic acid (AA, C20:4, ω-3). The lipid mediators, including thromboxane, prostacyclins or docsanoids, derived, respectively from AA, EPA and DHA, play important physiological roles in mammalian body. Our results are qualitatively consistent with other study (USDA 1988), where it was reported that bean contains both linoleic and linolenic acid. Some fatty acids are considered essential to mammals because of the fact that mammalian cells are unable to synthesize them. The essentiality of fatty acids was first described by Burr and Burr (1930). They identified ω-6 linoleic acid and alpha ω-3 linolenic acid as two essential fatty acids for growth, skin structural health, and reproduction in guinea pigs and rats. Since then, researchers have demonstrated the importance of polyunsaturated fatty acid as precursors of lipid mediator molecules, such as prostaglandins, prostacyclins, thromboxanes, leukotrienes, resolvins, neuroprotectins among others, which influence cellular functions. Polyunsaturated fatty acids are incorporated into phospholipids of cell membranes and influence structural and functional properties of cells. Polyunsaturated fatty acids, especially eicosapentaenoic acid (EPA) and docosahexaenoic acid (DHA), are being studied for their health benefits (Nair et al. 1997; Stone 1997; Caygill et al. 1996; Simopoulos 1999). Adequate DHA status is particularly important for infants (Oski 1997). α-Linolenic acid can be converted into EPA and DHA (Ghafoorunissa 1992). We have previously reported that EPA and DHA improve hypercholesterolemia, hypertension, platelet aggregation, oxidative stress and brain cognition in rats (Hashimoto et al. 1999, 2002, 2009, 2015; Hossain et al. 1995, 1998, 1999; Gamoh et al. 1999). Their roles in humans have also been reported elsewhere (Takayama et al. 2013; Hashimoto et al. 2012). The prime source of EPA and DHA is fish. However, vegetarians do not consume fish or meat, while meat/fish consumption among non-vegetarians from poorer social class is not adequate. Thus the α-linolenic acid, when consumed as plant-derived dietary source, might act as the precursor for the EPA and DHA in the vegetarians’ body. Furthermore, feeding fat to prepartum cows with unsaturated fatty acids seem to influence the pre-weaning growth of calves (José et al. 2013). In addition, the fatty acid composition of milk and other dairy products are of great interest both for the vegetarian and non-vegetarian consumers. Cow milk fat has been criticized due to its higher content of saturated fatty acid compared to vegetable fat or fish oil. Moreover, milk fat contains considerable amounts of myristic (C14:0) and palmitic acid (C16:0) and low concentrations of monounsaturated (MUFA) and polyunsaturated fatty acids (Kennelly 1996). The cow milk content of PUFAs can be increased by dietary feeding of oil seed, and the increase in PUFAs and the reduction of ω-6/ω-3 ratio of milk fat are desirable enrichment approaches for human health (Egger et al. 2007). Thus, the consumption of beans, through which ω3 α-linolenic acid intake can be increased, could be considered of significant interest. Polyunsaturated fatty acids can contribute to improve cardiovascular health and brain cognitions. The dietary ratio of ω-6 to ω-3 fatty acids among vegetarians (Messina and Messina 1996) is at the high end of the rather conservative recommendations by the World Health Organization (WHO). The ω-6 to ω-3 fatty acids ratio of *L. purpureus* in the present investigation was 4.86, indicating that the consumption of *L. purpureus* could be suggested for the vegetarians. In addition, beans contain both soluble and insoluble fibers, which might help to lower serum cholesterol in hypercholesterolemic individuals (Anderson et al. 1984) and several other diabetes risk factors (Salmerón et al. 1997). This report thus suggests that a host of beneficial effects might be obtained from *L. purpureus* seed powder.

Finally, the results of the proximate composition and fatty acid analyses of *L. purpureus* seed in this study demonstrate that the seed contains high nutrients with potentials to meet the nutritional requirements of human being. In addition to the outcome on the plasma proteins of malnourished rats, further research is underway to evaluate whether *L. purpureus* feeding can improve the levels of ω-3 polyunsaturated fatty acids (PUFAs) in the plasma, liver and brain tissues of these malnourished rats.
